# Emerging fungal threats from the environment—a lesson from *Candida auris* and a warning about a second candidate, *Rhodosporidiobolus fluvialis*

**DOI:** 10.1371/journal.ppat.1014356

**Published:** 2026-06-18

**Authors:** Yeseul Choi, Joseph Heitman, Eun Jeong Won

**Affiliations:** 1 Department of Molecular Genetics and Microbiology, Duke University Medical Center, Durham, North Carolina, United States of America; 2 Department of Laboratory Medicine, Asan Medical Center, University of Ulsan College of Medicine, Seoul, Republic of Korea; University of Michigan Health System, UNITED STATES OF AMERICA

## Introduction

Fungal infections have become an increasingly significant global health concern over the past several decades [1]. The emergence and spread of antifungal drug–resistant organisms have further constrained already-limited therapeutic options, posing substantial challenges to clinical management. Historically, invasive fungal infections have been attributed to a relatively limited group of well-characterized pathogens with the most prominent species within the genera *Candida*, *Aspergillus*, and *Cryptococcus* [2,3]. However, recent epidemiological trends indicate a marked shift in the spectrum of clinically relevant fungi.

In addition to healthcare-associated factors (such as increased immunocompromised patients, the use of immunosuppressive treatments, and invasive procedures), rapid climate changes are increasingly recognized as contributing factors in the emergence and redistribution of endemic fungal diseases [4–6]. Documented recent climate change leads to the concern that higher ambient temperatures will lead to the selection of fungal lineages that have become more thermally tolerant, such that they can breach the mammalian thermal restriction zone [7]. Several nonpathogenic fungal species, particularly those found in soil or the environment, possess the capacity to adapt to higher temperatures, which may enhance their ability to survive in human hosts and acquire virulence factors [8].

*Candida auris* is one example emerging as a significant pathogen around the globe associated with adaptation to higher temperatures. It is interesting that this pathogen was not new, and it likely existed in the environment long before its recognition as a human pathogen. Likewise, proactive efforts to identify fungi that are ready to be potential pathogens are warranted to enable early detection, characterization, and mitigation before widespread clinical impact. As part of a study seeking to identify novel pathogenic fungi, researchers collected samples from patients in 96 hospitals in China between 2009 and 2019 [9]. Among 27,100 fungal strains collected, one had never been documented to infect humans before: the yeast *Rhodosporidiobolus fluvialis*. It was isolated from the blood of two patients treated in different intensive care units for severe underlying diseases, and these two patients died despite receiving fluconazole and caspofungin therapy. Importantly, only two isolates of *R. fluvialis* were identified in this large-scale surveillance study, underscoring the apparent rarity of this organism in current clinical settings. Although this rare, thermotolerant, multidrug-resistant fungus may be an emerging pathogen, little is known about it. Herein, we summarize relevant features of *R. fluvialis* and compare these properties to those of the well-known emerging pathogen, *C. auris*.

## From the environment to the host

**Fig 1:**
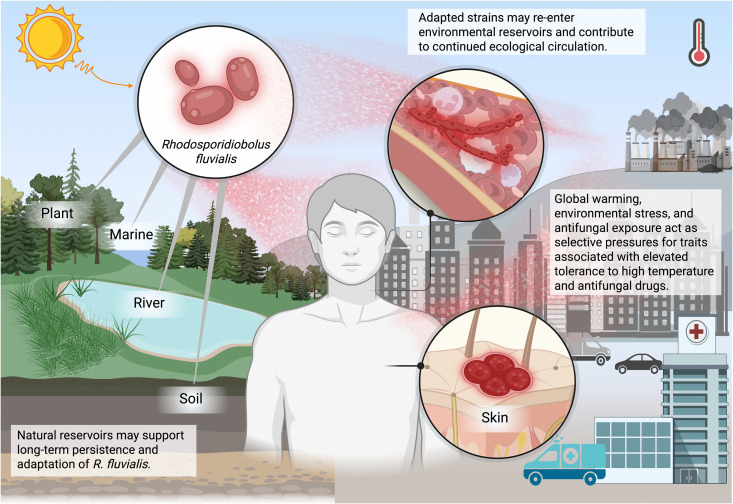
Proposed environmental-to-clinical transition of *R. fluvialis.* *R. fluvialis* is an environmental yeast found in diverse ecological niches, including water, soil, and plant-associated habitats. Environmental pressures such as global warming, other stresses, and antifungal exposure may act as selective forces favoring thermotolerant and stress-adapted populations. These adapted strains may increase the likelihood of human exposure, potentially enabling transient colonization, including possible skin-associated persistence, and entry into human-associated environments. Under certain conditions, this may lead to opportunistic invasive infection, including bloodstream infection. Once adapted to human-associated niches, strains may circulate between environmental and clinical settings, contributing to their continued persistence and potential emergence as human pathogens. Citation to Use: Created in BioRender. Choi, Y. (2026) https://BioRender.com/rcxa5hk.

*Rhodosporidiobolus fluvialis* (previously known as *Rhodosporidium fluviale*) was first isolated in 1972 from a water sample collected from the Miami River, Miami, Florida [10]. It is assigned to the family of Sporidiobolaceae, order of Sporidiobolales, class of Pucciniomycotina, and phylum of Basidiomycota in the Fungal kingdom as recorded by the International Mycological Association [11]. The genus *Rhodosporidiobolus* was recently proposed by Wang et al. [11] and includes nine species: *Rhodosporidiobolus fluvialis, R. azoricus, R. microsporus, R. nylandii, R. ruineniae, R. lusitaniae, R. colostri, R. odoratus*, and *R. poonsookiae* [12]. These species have been isolated from soil, plants, freshwater, or litter [13–16] (Fig 1). Notably, *R. fluvialis* has been reported in water (including from contaminated or polluted areas), a marine sponge, and the phylloplane of corn plants [8,17,18]. These environmental associations may suggest adaptation to aquatic or saline-associated niches, reminiscent of the halotolerance and marine association described for *C. auris* [19,20]. Given that halotolerance and environmental persistence are thought to contribute to the emergence and transmission of *C. auris*, it will be important to determine whether similar traits are present in *R. fluvialis*.

Although *R. fluvialis* had not been reported in the human GI tract by culture-dependent investigations [21], members of the order Sporidiobolales, including *Rhodotorula*, *Rhodosporidium*, and *Sporobolomyces*, were detected at relatively high abundance in the skin mycobiome of patients with diffuse systemic sclerosis [22]. While this does not directly demonstrate *R. fluvialis* skin colonization, these observations raise the possibility that related Sporidiobolales yeasts may persist on human skin (Fig 1). In contrast, *C. auris* is well recognized for its ability to colonize human skin despite not being considered a commensal yeast and rarely being detected on mucosal surfaces or in the GI tract [23]. The skin tropism is thought to contribute to healthcare-associated transmission, particularly when combined with its ability to form biofilms and persist on abiotic surfaces, including plastics and medical devices. Notably, *C. auris* can survive for prolonged periods on hospital-associated surfaces and exhibits tolerance to several commonly used disinfectants, features that likely contribute to its remarkable nosocomial persistence and global spread [23]. Whether *R. fluvialis* possesses similar capacities for biofilm formation, environmental persistence, or disinfectant tolerance remains unknown, and future studies investigating these traits may help explain the currently limited number of reported clinical isolates and the apparent lack of widespread healthcare-associated transmission.

## Morphological characteristics and identification

Morphologically, colonies of *R. fluvialis* are light scarlet orange, smooth, glistening, slightly raised with a complete border. They can form intracellular lipid droplets full of carotenoids, which makes them range in color from pink to orange in culture media. These morphological characteristics are typical for red yeasts of the genera *Rhodosporidiobolus* [9]. *R. fluvialis* grows predominantly as a yeast with a small proportion of cells showing pseudohyphal morphology. Microscopically, budding cells are spherical to ellipsoidal and bud scars are narrow. Ballistoconidia may be present and hyphal elements are occasionally produced. Data on the pathophysiology of this fungus are scarce, with just two cases of bloodstream infection caused by *R. fluvialis* reported in the literature to date [9].

Although commercial MALDI-TOF MS systems, the Vitek MS and the Bruker Biotyper MS, have been widely utilized for the identification of yeasts, *R. fluvialis* has not yet been included in either database. Therefore, MS-based identification approaches are not yet useful as diagnostic tools for infections caused by *R. fluvialis* [24]*.* Internal transcribed spacer (ITS) sequencing can be employed to correctly identify these isolates to the species level; however, it is not practical for a timely diagnosis. Initially, *C. auris* also faced similar challenges in conventional identification based on biochemical platforms; e.g., the API 20C AUX and API ID 32C systems that often reported isolates as *Rhodotorula glutinis*, *Saccharomyces cerevisiae*, or *Candida sake*, while the VITEK 2 system misclassified them as *C. haemulonii* [25–28]. Given these limitations, the CDC recommends that biochemical test results always be interpreted with caution and in conjunction with confirmatory diagnostics, such as MALDI-TOF MS or DNA sequencing [29].

## Thermotolerance and host adaptation

Increasing global temperatures are proposed to drive adaptation, enabling fungi to grow at higher temperatures or favoring the selection of fungi with enhanced thermotolerance [30]. It has been hypothesized that environmental adaptation in response to rising global temperatures contributed to the emergence of pathogenic *C. auris* [31,32]. *C. auris* flourishes at 42 °C, making thermal tolerance a distinguishing feature of *C. auris* compared with other *Candida* species [23,31]. Additionally, it has been reported that there is a variation of thermotolerance in different clades of *C. auris*, in that clade II isolates are less thermotolerant than non-clade II isolates [33]. It makes sense based on the unique characteristic of clade II *C. auris* isolates derived from the ear, which is cooler than core body temperature [33,34]. Rather, the superior thermotolerance of non-clade II isolates might enable them to endure on warmer skin regions such as the axilla and groin, which are common *C. auris* isolation sites. Collectively, thermal adaptation facilitates colonization of specific niches and aids in the prolonged environmental persistence of nonclade II isolates of *C. auris,* ultimately enabling it to be more pathogenic to humans.

It is noteworthy that *R. fluvialis* is also thermotolerant, in contrast to other *Rhodosporidiobolus* strains [9]. In a previous study, mice infected with *R. fluvialis* showed high fungal burden in multiple organs, indicating a strong capacity for in vivo adaptation in the mouse model. The exact mechanism of thermotolerance of this fungus is unknown. So far, several attributes have been shown to be involved in heat-resistance, including heat shock proteins (Hsps), trehalose, ATPase, ubiquitin, and antioxidant enzymes, all of which play important roles in yeast heat-resistance mechanisms. Heat shock proteins (Hsps) enable organisms to resist high temperatures. Hsps protect thermally damaged proteins from aggregation, promote refolding damaged proteins, clear irreversibly aggregated proteins, and improve the thermal stability of soluble proteins, SOD, and proton pumps in stressed cells [35]. In addition, the calcineurin-Crz1 pathway could play a critical role in environmental adaptation in yeast and various fungal pathogens, and several researchers reported that the calcineurin pathway is essential for stress adaptation and virulence, particularly under high-temperature conditions, in *Saccharomyces cerevisiae, Cryptococcus neoformans, Candida albicans*, and *Candida glabrata* [36–39]. Recently, Cha et al. [40] reported that the calcineurin pathway also regulates extreme thermotolerance, antifungal resistance, and virulence in *C. auris*. Future research should focus on the elucidation of thermotolerance mechanisms in *R. fluvialis*, which may provide insights into the evolutionary path that enabled human infection.

## Morphological plasticity and pseudohyphae formation

**Fig 2:**
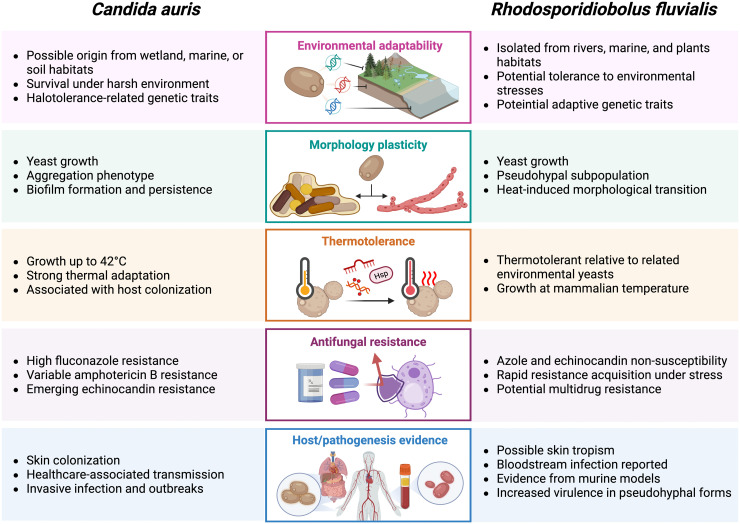
Comparison of key traits associated with fungal emergence in *C. auris* and *R. fluvialis.* *C. auris*, an established emerging pathogen, exhibits thermotolerance, antifungal resistance, and the ability to colonize the skin and cause systemic infection, facilitating healthcare-associated transmission. In contrast, *R. fluvialis* is an environmental yeast with emerging pathogenic potential, sharing several features including thermotolerance, reduced antifungal susceptibility, and morphological plasticity, such as pseudohyphal formation. To date, only two cases of bloodstream infection have been reported; however, limited clinical and experimental evidence suggests a potential for invasive disease. Together, these comparisons highlight shared and distinct traits that may contribute to the emergence of environmental fungi as human pathogens. Citation to Use: Created in BioRender. Choi, Y. (2026) https://BioRender.com/87tc422.

During human infection, fungi can undergo a dimorphic transition from yeast cells to elongated filaments to adapt to various conditions within the host. This allows exploration of new environments and tissue invasion and is considered a virulence trait in pathogenic fungi [41–43]. *C. auris* grows as yeast cells (without pseudohyphae) in an aggregating and nonaggregating form, which present differences in virulence, resistance to disinfectants, and biofilm formation [44,45] (Fig 2). Aggregation is increased at higher growth temperature, suggesting a possible link with thermotolerance and virulence. However, aggregating *C. auris* isolates were found to be less virulent than nonaggregating isolates in an invertebrate infection model [46]. Instead of aggregation*, R. fluvialis* exhibits a minor population of cells that form pseudohyphae*,* and this results in wrinkled colonies [9] (Fig 2). Pseudohyphal mutants have also been generated via heat stress induction and found to harbor mutations in Ace2. Pseudohyphae formation in *R. fluvialis* was found to be related to worse prognosis in the murine model, partly explained by the inadequate internalization and a significant increase in macrophage lysis by these pseudohyphae forming mutants. These findings highlight how heat stress might stimulate morphological plasticity, resulting in increased virulence of this fungus.

## Antifungal resistance and possible mechanisms

The emergence of new pathogenic fungi becomes particularly concerning when adaptation is accompanied by reduced antifungal susceptibility. *C. auris* exhibits resistance to fluconazole and variable susceptibility to other azoles, amphotericin B, and echinocandins [23,34,47]. Approximately 87–100% of *C. auris* isolates are resistant to fluconazole, up to 35% are resistant to amphotericin B, and 0–8% are resistant to echinocandins. Several antifungal resistance mechanisms of *C. auris* have been addressed, such as mutations in hot spots of the target gene *ERG11*, over-expression of efflux pumps (*CDR1*, *CDR2*, and *MDR1*), gain of function mutations in a transcription factor, gene duplication, modifications in lipid content, or biofilm formation, respectively [48–52]. A previous study reported that Tac1B mutations were the predominant fluconazole resistance mechanism in clade II isolates [33], and suggested that the combined presence of both *ERG11* and *TAC1B* mutations could have a cumulative effect, resulting in elevated fluconazole MIC values, similar to *C. albicans* [53,54]. One of the reasons why the emergence of *R. fluvialis* is alarming is the potential for this species to harbor resistance to fluconazole and echinocandins, and it even has the capacity to develop multidrug resistance [9]. The duplication of *ERG11* genes and over-expression of *ERG11* were suggested as a resistance mechanism to multiple azole regimens, and carotenoid production could lead to caspofungin resistance [9]. The optimal treatment for *R. fluvialis* has not been defined yet, owing to these factors and the small number of clinical cases reported to date. Considering innate resistance to azoles and echinocandins, amphotericin B could be initial treatment option. It was noteworthy that *R. fluvialis* could rapidly develop 5-fluorocytosine-resistance, even though it was initially sensitive to 5-fluorocytosine [9]. Huang et al. reported that Amphotericin B was generally effective in eradicating *Rhodosporidiobolus* cells, but incubation at 37 °C could induce the emergence of amphotericin B-resistant mutants in different *Rhodosporidiobolus* species [9]. Collectively, their thermotolerance could be linked to the gain of multidrug resistance or even pan-drug resistance, finally resulting in limiting available therapeutic options clinically (Fig 2).

## Conclusions and future directions

Environmental conditions are continuously evolving, and the selective pressures imposed by climate change, antifungal exposure, and shifting host demographics may increasingly favor fungi capable of adapting to human-associated niches. Recent examples, most notably *C. auris*, demonstrate that environmental or previously unrecognized species can rapidly acquire traits that render them more difficult to treat and potentially more deadly than established pathogens. Although *R. fluvialis* currently appears to be an exceedingly rare clinical isolate, its recent association with human infections and its thermotolerant, multidrug-resistant features warrant further attention. In addition to thermotolerance, *R. fluvialis* has several traits such as nonsusceptible to antifungal agents and morphological changes that enhance their ability to invade or persist within the hosts (Fig 2). Whether *R. fluvialis* possesses the same degree of skin colonization, environmental persistence, and healthcare-associated transmission capacity as *C. auris* remains unknown. Notably, the potential reentry of adapted strains into environmental reservoirs may accelerate their spread and increase emergence risk (Fig 1). Furthermore, the potential impact of climate change on the emergence of pathogenic fungi, causing them to adapt to hotter temperatures, enabling them to infect humans, highlights the need for increased surveillance and research on these emerging pathogenic threats.
